# Geographical clustering of delayed motherhood in India: an application of multilevel and geospatial analyses

**DOI:** 10.3389/fgwh.2026.1762981

**Published:** 2026-08-17

**Authors:** Mayank Singh, Chander Shekhar, Jagriti Gupta, Rajeev Kumar

**Affiliations:** 1 Department of Epidemiology and Biostatistics, KLE Academy of Higher Education and Research, Belagavi, Karnataka, India; 2 Department of Fertility & Social Demography, International Institute for Population Sciences (IIPS), Mumbai, India; 3 Biostatistician, Bagchi School of Public Health, Ahmedabad University, Ahmedabad, Gujrat, India; 4 PG Department of Statistics, Patna University, Patna, Bihar, India

**Keywords:** age at marriage, delayed motherhood, district-level disparities, India, maternal and child health, multilevel analysis, socioeconomic determinants, spatial analysis

## Abstract

**Background:**

Rapid sociodemographic changes in India, including rising female education, workforce participation, urbanization, and changing gender norms, have contributed to delayed childbearing. Despite declining fertility, limited evidence exists on the district-level geographic variations and determinants of delayed motherhood (childbirth at age ≥35) in India. This study applies multilevel and geospatial analyses to examine the distribution, determinants, and district-level spatial clustering of delayed motherhood using National Family Health Survey data.

**Methods:**

This study employed multilevel multivariable logistic regression to examine individual- and district-level determinants of delayed motherhood. Spatial clustering was assessed using Global Moran's *I* and Local Indicators of Spatial Association (LISA). To account for spatial dependence across districts, a comparative spatial modeling framework was applied using ordinary least squares regression as the baseline model, followed by the spatial lag model and the spatial error model.

**Results:**

The prevalence of delayed motherhood varied widely, ranging from negligible levels in some districts to as high as 60.18% in South West Khasi Hills, with a national average of 8.7% [95% confidence interval (CI): 8.1–9.3]. Higher education [adjusted odds ratio (AOR): 2.00; 95% CI: 1.80–2.23] and age at marriage ≥21 years (AOR: 5.13; 95% CI: 4.65–5.67) were significant predictors. Additional factors such as higher parity, religious affiliation, and socioeconomic status also showed significant associations. A spatial analysis revealed strong clustering (Moran's *I* = 0.459), with high–high clusters in southern and northeastern regions and low–low clusters in central and northern India. Spatial models confirmed significant spillover effects across neighboring districts.

**Conclusion:**

Delayed motherhood in India exhibits substantial sociodemographic and spatial heterogeneity. Key determinants include education, marriage timing, and parity patterns. The presence of distinct geographic clusters highlights the importance of regional context. These findings underscore the need for region-specific and demographically targeted policy interventions, along with further research into behavioral and health system factors influencing delayed childbearing.

## 1 Background

Delayed motherhood has emerged as a critical demographic and public health issue globally, often interpreted through the lens of demographic transition theory and the second demographic transition (SDT) framework, which link postponement of first birth to rising female education, urbanization, labor force participation, economic modernization, and changing gender norms (1–3). In many high-income countries, delayed childbearing has been associated with shifts toward individualism, expansion of higher education, and women's increased autonomy in reproductive decision-making (4, 5). However, the trajectory of delayed motherhood in India reflects a more complex and uneven transition shaped by entrenched early marriage practices, sociocultural heterogeneity, caste and class stratification, regional disparities, and differential pace of socioeconomic development (6–8). Unlike Western contexts where fertility postponement emerged after substantial socioeconomic transformation, India exhibits the coexistence of traditional and modern reproductive regimes operating simultaneously across states and districts.

Over the past three decades, India has experienced sustained fertility decline accompanied by gradual increases in the mean age at marriage and age at first birth (9, 10). Yet, these improvements have been uneven across regions, reflecting stark interstate and intrastate disparities in female education, urbanization, labor market opportunities, and access to reproductive health services (9, 10). Empirical evidence from large-scale nationally representative surveys underscores the importance of contextual and structural determinants in shaping reproductive timing. For instance, district-level multilevel and geospatial analyses of 707 districts in India documented substantial clustering of early marriage and early motherhood, demonstrating that socioeconomic disadvantage, limited female schooling, and contextual vulnerabilities significantly influence reproductive transitions (11). Complementing this, recent joinpoint and age–period–cohort analyses have revealed cohort-driven increases in delayed motherhood at the national level, suggesting generational shifts in fertility timing linked to educational expansion and socioeconomic mobility (12). These findings collectively indicate that while national trends point toward gradual postponement, subnational heterogeneity remains pronounced.

India's rich demographic data infrastructure provides multiple sources for examining fertility timing and its determinants. The National Family Health Survey (NFHS), conducted under the stewardship of the International Institute for Population Sciences (IIPS), offers detailed district-level data on fertility, age at marriage, maternal health, and socioeconomic indicators (13). The sample registration system supplies reliable annual estimates of fertility and mortality; the District-Level Household and Facility Survey (DLHS) provides insights into maternal and reproductive health service utilization; the Longitudinal Ageing Study in India (LASI) captures life-course and intergenerational dynamics; and the National Sample Survey (NSS) offers complementary socioeconomic information. Together, these datasets enable a comprehensive examination of reproductive behavior within broader developmental contexts. Nevertheless, despite the availability of these extensive datasets, relatively few studies have explicitly integrated spatial econometric approaches to analyze geographic clustering and spatial dependence in delayed motherhood across districts.

Understanding delayed motherhood in India also requires attention to policy and institutional contexts. National programs such as the National Health Mission, expansion of secondary and higher education for girls, conditional cash transfer schemes promoting delayed marriage, and enforcement of the Prohibition of Child Marriage Act (2006) have contributed to shifts in reproductive timing (14, 15). However, the implementation and effectiveness of these policies vary widely across states because of differences in governance capacity, administrative efficiency, and sociocultural acceptance. Consequently, aggregate national averages often mask localized pockets of vulnerability and resilience (9). Persistent gender inequality, patriarchal kinship systems, and economic insecurity continue to shape reproductive choices in certain regions, while metropolitan and economically advanced districts increasingly reflect aspirations for career development and delayed family formation (5, 8).

Given India's demographic diversity and spatial inequality, examining delayed motherhood through a spatially informed framework is particularly relevant. Districts do not operate as isolated demographic units; rather, they are embedded within regional systems characterized by shared socioeconomic environments and diffusion of norms. By situating delayed motherhood within India's unique sociocultural, economic, and policy landscape and leveraging nationally representative datasets alongside spatial analytical techniques, there are also some population-based consequences of delayed motherhood. Delayed motherhood causes a larger generation gap; women and men are less likely to see their grandchildren who initiated parenthood at a later age as compared to women and men who initiated their parenthood at an early age (16, 17). Women who would have a child in a given year shifted their pregnancy in the future, their period fertility rate was depressed and fell below the corresponding cohort family size indicator, and it negatively affected period birth rates (18). In low-mortality countries, when fertility rates are below replacement level, delayed motherhood also leads to a slower decline in population because of the generational gap (19). There is a limited amount of research on delayed motherhood, especially in the context of India. Therefore, in this article, our study aims to observe the distribution of delayed motherhood in India, and in order to observe the factors that influenced delayed motherhood in a multilevel context and also to note spatial heterogeneity and mesoscale correlates of delayed motherhood across 707 districts of India, this study seeks to advance understanding beyond generalized global narratives. It provides a nuanced assessment of how modernization, gender transformation, and regional inequality intersect across space to shape reproductive timing in contemporary India.

## 2 Materials and methods

### 2.1 Data source

The latest round of the National Family Health Survey (NFHS-V), 2019–21, popularly known as the Demographic Health Survey (DHS), has been used as a data source. This survey collects the data for 707 districts nested within 36 states and union territory, covering 724,115 eligible women in the age group 15–49 years. A two-stage stratified sampling design in urban and rural areas was used. In addition, for the selection of primary sampling units, i.e., a village in rural areas and a census enumeration block in urban areas, the 2011 census served as a sampling frame. The NFHS contains detailed information about the age at occurrence of reproductive events such as age at menarche, age at first marriage, age at first cohabitation, age at first sex, and age at first birth and latest birth. This survey also includes detailed information on demographics, family planning, nutrition, maternal and child health, reproductive outcomes, and other maternal health issues. The present study used national as well as district-level estimates of delayed motherhood to observe their predictors and spatial heterogeneity across 707 districts provided by NFHS 2019–2021 (20).

#### 2.1.1 Sample selection

**Figure 1:**
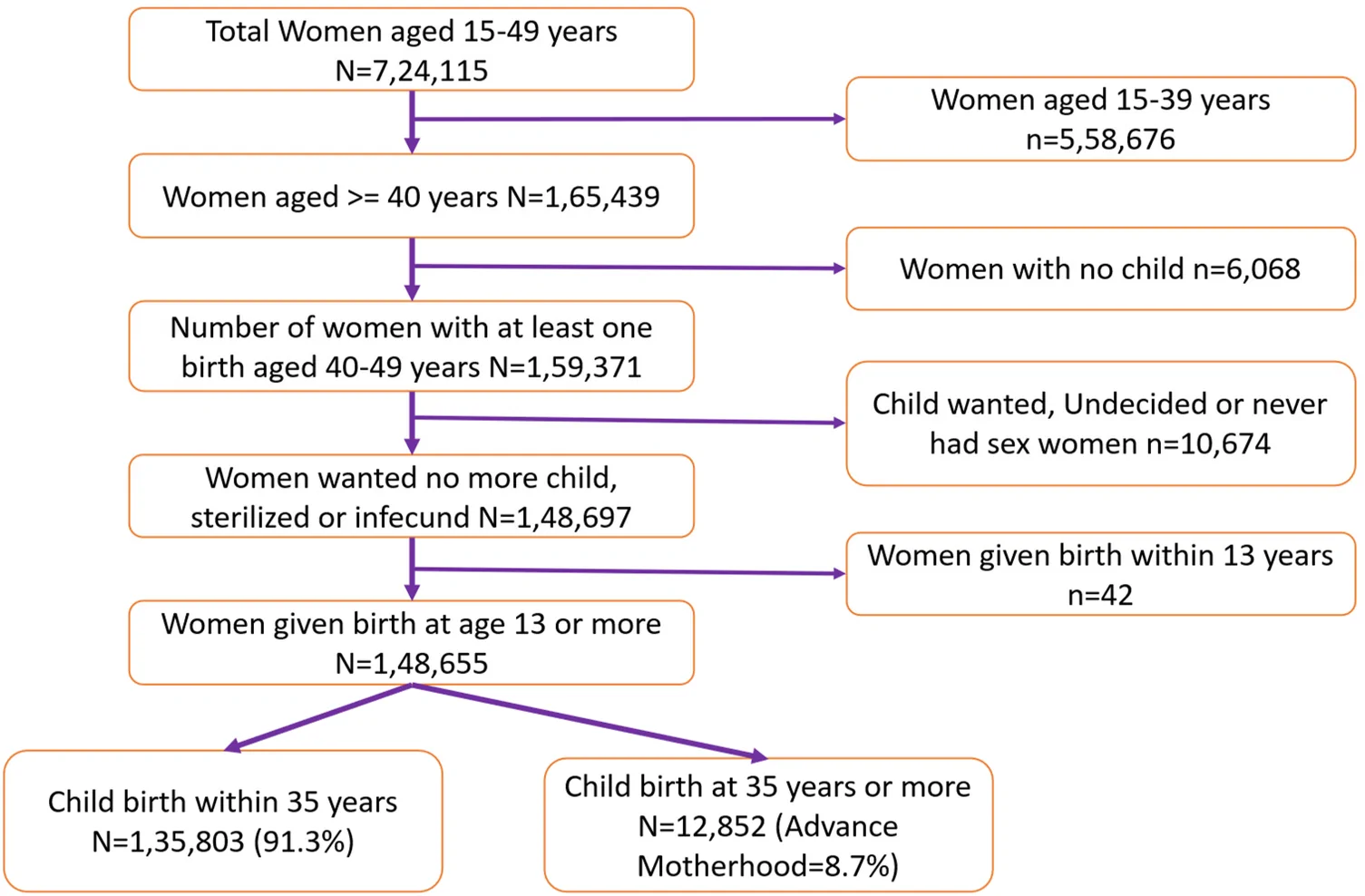
A flow diagram depicting the sample selection process for delayed motherhood in India 2019–21.

The study utilized data from the National Family Health Survey-5 (NFHS-5), 2019–2021, which interviewed 724,115 women aged 15–49 years across India (19). For the present analysis, women aged 15–39 years (*n* = 558,676) were excluded to ensure near-complete fertility exposure and to increase the likelihood that the most recent birth represented the last birth. This resulted in 165,439 women aged 40 years and above. Among them, women with no children (*n* = 6,068) were excluded. Furthermore, women who reported that they wanted more children, were undecided about future childbearing, or never had sexual intercourse (*n* = 10,674) were excluded to focus on women with completed or near-completed fertility. In addition, women who reported a birth within 13 years preceding the survey (*n* = 42) were excluded. After applying these exclusions, the final analytical sample consisted of 148,655 women aged 40–49 years with at least one live birth. Delayed motherhood was defined based on the age at the most recent childbirth, which likely represents the last birth in this completed fertility cohort. Women whose last birth occurred at age ≥35 years were classified as experiencing delayed motherhood (*n* = 12,852; 8.7%), while those whose last birth occurred before age 35 constituted the reference group (*n* = 135,803; 91.3%) (Figure 1). This classification was independent of birth order; delayed motherhood was determined solely by maternal age at the most recent childbirth rather than parity.

### 2.2 Variable description

#### 2.2.1 Outcome variable

During the survey, respondents were asked to provide the month and year of birth for each of their children. This information was used to calculate the exact maternal age at each childbirth, including the most recent birth. Maternal age at last (most recent) childbirth was derived by subtracting the respondent's date of birth from the reported month and year of the child's birth. The outcome variable, delayed motherhood, was constructed as a dichotomous variable and defined as having the most recent childbirth at age ≥35 years (“yes”) or <35 years (“no”), irrespective of parity (21). Using this classification, the study examined the associated factors related to delayed motherhood, and furthermore, district-level estimates were used for a spatial analysis.

#### 2.2.2 Explanatory variables

Socioeconomic and demographic covariates included in the study were categorized as follows. The place of residence was taken as rural and urban, as provided in the survey. Religion was recorded as Hindu, Muslim, Christian, and Others. Ethnicity (Caste) was recoded as SC, ST, OBC, and others. The Wealth Index was coded as Poorest, Poorer, Middle, Richer, and Richest. The educational status of respondents was recoded as No education, Primary, Secondary, and Higher. Mass Media exposure was coded as Low, Moderate, and High for the questions (i) Do you read a newspaper or magazines almost every day, at least once a week, less than once a week or not at all? (ii) Do you listen to the radio almost every day, at least once a week, less than once a week, or not at all? (iii) Do you watch television almost every day, at least once a week, less than once a week, or not at all? (iv) Do you usually go to a cinema hall or theater to see a movie at least once a month? Regions of India were coded as East, West, North, South, Central, and Northeast. Relation to husband prior to marriage was coded as yes and no. In addition, previous parity was recoded as zero, one-two, three-four, and five more. Age at first marriage and first sex were coded as <18, 18–21, and ≥21 years.

### 2.3 Statistical analysis

The analysis began with descriptive statistics to present the frequency distribution of respondents and the prevalence of delayed motherhood. A bivariate analysis using the chi-square test was conducted to examine the association between delayed motherhood and selected sociodemographic characteristics. A *P*-value of <0.05 was considered statistically significant. To assess potential multicollinearity among explanatory variables, the variance inflation factor (VIF) was computed for all covariates included in the regression models. The VIF values were within acceptable limits (VIF < 10), indicating no evidence of multicollinearity (22).

#### 2.3.1 Model specification for multilevel logistic regression

A four-level multilevel logistic regression model was employed to examine variations in delayed motherhood across hierarchical geographic levels. The data structure comprised individuals (level 1) nested within primary sampling units (PSUs) (level 2), which were nested within districts (level 3), and further nested within states/union territories (level 4).

Let $Y_{ijkl}$ denote the delayed motherhood status of woman *i*, residing in PSU *j*, district *k*, and state *l*, defined as follows:

$$Y_{ijkl}=\{\begin{array}{cc} 1, & \mathrm{if}\;\mathrm{the}\;\mathrm{most}\;\mathrm{recent}\;\mathrm{birth}\;\mathrm{occurred}\;\mathrm{at}\;\mathrm{age}\;\geq 35\\ 0, & \mathrm{if}\;\mathrm{the}\;\mathrm{most}\;\mathrm{recent}\;\mathrm{birth}\;\mathrm{occurred}\;\mathrm{before}\;\mathrm{age}\;35 \end{array}$$

The probability of experiencing delayed motherhood was modeled using a four-level random-intercept logistic regression model specified as follows:

$$\mathrm{Logit}\;[P(Y_{ijkl}=1)=\;\beta_{0}+\;\;\sum_{\;p=1}^{P}\beta_{p}X_{\;pijkl}+(\;u_{0l}+\;u_{0kl}+\;u_{0jkl}\;)$$

where $\beta_{0}$ is the overall fixed intercept, representing the log-odds of delayed motherhood for women in the reference category of all explanatory variables, $X_{\;pijkl}$ is a vector of individual and household level covariates (e.g., maternal age, parity, education, wealth status, antenatal care utilization, and place of residence), $\beta_{p}$ are the corresponding fixed-effect coefficients, $u_{0jkl}∼N(0,\sigma_{u}^{2})$ represents the random effect at the PSU level, $v_{0kl}∼N(0,\sigma_{v}^{2})$ represents the random effect at the district level, and $f_{0l}∼N(0,\sigma_{f}^{2})$ represents the random effect at the state/union territory level**.** These random intercepts capture unobserved contextual heterogeneity operating at different geographic levels.

##### 2.3.1.1 Four nested multilevel logistic regression models were fitted

*Model I (Null Model)*: Included only random intercepts to assess the extent of clustering in delayed motherhood across geographic levels and to compute the intraclass correlation coefficient (ICC).

*Model II*: Included individual-level characteristics.

*Model III*: Included both individual- and household-level characteristics.

*Model IV (Full Model)*: Included individual, household, and community-level variables.

The ICC was calculated using the latent variable approach for logistic regression models:

$$\mathrm{ICC}=\frac{\sigma_{u}^{2}+\sigma_{v}^{2}+\sigma_{f}^{2}}{\sigma_{u}^{2}+\sigma_{v}^{2}+\sigma_{f}^{2}+\frac{\pi^{2}}{3}}$$

where $\pi^{2}/3=3.29$ represents the individual-level variance of logistic distribution. The model fit was assessed using the Akaike information criterion (AIC) and likelihood ratio (LR) tests. The model with the lowest AIC was considered the best-fitting model. All models were estimated using maximum likelihood estimation, and the results are presented as adjusted odds ratios (AORs) with 95% confidence intervals (CIs) (23–25).

#### 2.3.2 Statistical specification for the spatial analysis

To assess geographic clustering and spatial dependence of delayed motherhood, Global Moran's *I*, Local Indicators of Spatial Association (LISA), and significance maps were employed. Global Moran's *I* was used to examine whether delayed motherhood exhibited spatial autocorrelation across districts. Moran's *I* ranges from −1 to +1, where positive values indicate clustering of similar values (high–high or low–low), negative values indicate spatial dispersion, and values near zero suggest a random spatial pattern (26, 27). Univariate LISA maps were generated to identify district-level clusters of delayed motherhood. Bivariate LISA maps were further used to assess the spatial association between delayed motherhood and selected district-level predictors by examining the relationship between the outcome in a given district and the spatially weighted average of explanatory variables in neighboring districts (27, 28).

*Ordinary Least-Squares (OLS) Model*: The OLS model serves as a baseline for comparison in spatial analyses when spatial dependence is a concern (29). Initially, an OLS model was fitted to identify the significant district-level determinants, as a baseline specification:

Y=Xβ+ε

where *Y* denotes district-level prevalence of delayed motherhood, *X* represents explanatory variables, *β* indicates regression coefficients, and $\varepsilon ∼N(0,\sigma^{2}I)$ is the independently distributed error term. Spatial dependence in the residuals was assessed using Moran's *I* (26). As the OLS residuals exhibited significant spatial autocorrelation, spatial regression models were subsequently estimated.

*Spatial Lag Model*: The spatial lag model (SLM) is designed to explicitly account for spatial dependence in the dependent variable itself. It incorporates a spatially lagged dependent variable, capturing spillover effects where the outcome in one district is influenced by the outcomes in neighboring districts (30). The SLM was specified as follows:

Y=ρWY+Xβ+ε

where *ρ* is the spatial autoregressive coefficient, which typically falls within the range of −1 to 1, indicating the strength and direction of the spatial dependence. W is the spatial weights matrix, a critical component that defines the neighborhood relationships between spatial units (26, 30). This matrix can be row-standardized and is often constructed based on contiguity (e.g., shared borders) or inverse distance, meaning that closer units exert a greater influence. The term *WY* represents the spatially lagged dependent variable, signifying the average value of the dependent variable in neighboring districts. The error term *ε* maintains the assumption of i.i.d. normal distribution, similar to the OLS model. The SLM is particularly useful when the dependent variable in a given location is influenced by the dependent variable in adjacent locations. Researchers have also proposed modifications to SLM estimation, especially in scenarios with missing data, including two-stage least squares with imputation and generalized non-linear least squares. More advanced forms such as semiparametric and semivariable coefficient spatial lag models have been developed to address complex spatial relationships and non-linearities (29–33).

*Spatial Error Model*: The spatial error model (SEM) addresses spatial dependence present in the error terms of the regression model, indicating unobserved spatial processes that affect the outcome (29). This implies that factors not included in the model, but which are spatially correlated, are influencing the dependent variable. The SEM can be expressed in either of the following two forms:

Y=Xβ+uu=λWu+ε

OR

$$Y=X\beta +(I-\lambda W{)}^{-1}\varepsilon$$

Here, λ is the spatial autoregressive parameter for the error term *u*, quantifying the spatial autocorrelation in the residuals (29). *W* is again the spatial weights matrix, defining neighborhood relationships. The term *u* represents the spatially correlated error component, and ε is the i.i.d. error term. The SEM is suitable when spatially correlated omitted variables or measurement errors are suspected to influence the dependent variable (29, 34).

When choosing between the SLM and the SEM, or determining their suitability over OLS, model performance is typically evaluated using criteria such as the AIC and log-likelihood values (29). A lower AIC value and a higher log-likelihood value generally indicate a better model fit, suggesting that the chosen spatial model more adequately captures the underlying spatial processes than a non-spatial model like OLS. The application of these spatial models is crucial for understanding the true impact of factors like unhygienic menstrual hygiene practices on child undernutrition at a district level, especially when geographic proximity leads to correlated outcomes (29). All analyses accounted for survey weights where appropriate. Statistical analyses were conducted using STATA, while spatial analyses were performed using GeoDa (28).

## 3 Ethical consideration

The study used publicly available NFHS-5 data. Informed consent was obtained from all participants by the survey agencies during data collection, and respondents' privacy and confidentiality were strictly maintained. The NFHS survey protocol, including all questionnaires, was approved by the Institutional Review Board (IRB) of the International Institute for Population Sciences (IIPS) and the ICF Institutional Review Board. The protocol was also reviewed by the U.S. Centers for Disease Control and Prevention (CDC). Permission to use the dataset was obtained from the Demographic and Health Surveys (DHS) Program, and the data are available in the public domain. Therefore, no additional ethical approval was required for this secondary data analysis.

## 4 Results

Table 1 depicts the univariate and bivariate analyses showing the weighted distribution of delayed motherhood by background characteristics. In the study, a total of 148,655 women aged 40–49 years were included. Among them, about 21.7% of respondents belong to the central region, followed by 20.1% belonging to the northern region. Nearly two-third (65.6%) of the respondents were rural residents, and nearly four out of ten respondents (44.2%) were illiterate. The majority (83.1%) of the respondents were Hindu, and less than half of the respondents (48.6%) were married before 18 years of age. Close to three-fifths (60.3%) of the respondents were already having 1–2 children before their last birth. The overall rate of prevalence of delayed motherhood among women aged 40–49 was found to be 8.7%. In terms of the respondent's background characteristics, the highest prevalence (19.7%) of delayed motherhood was found in the northeastern part of India, followed by 13.2% and 13.1% in the eastern and central parts of India. The prevalence of delayed motherhood makes a U-shape pattern with the educational background. Approximately 1 out of 10 women belonging to the Scheduled Tribe caste give their last childbirth after the age of 35 years. The prevalence of delayed motherhood was highest among Muslims (15.3%), followed by Christians (11.3%). The prevalence of delayed motherhood also varied across subgroups of age at first marriage and age at first sex, from 5.8% (5.9%) among women married (sex) below 18 years to 16% (15.9%) among women married (sex) after 20 years. The result also shows a 31.4% difference in the prevalence of delayed motherhood by the previous parity subgroup, ranging from 4.7% among 0 previous parity to 36.1% among 5+ previous parity.

**Table 1:** Univariate and bivariate analyses showing the weighted distribution of delayed motherhood by background characteristics.

Characteristics	Total		Delayed Motherhood (Yes): N (%)	Chi-square (*P*-value)
	*N*	Percentage		
Regions				
East	22,690	15.3	2,985 (13.2)	<0.001
West	16,200	10.9	926 (5.7)	
North	29,876	20.1	2,314 (7.8)	
South	27,704	18.6	1,157 (4.2)	
Central	32,266	21.7	4,221 (13.1)	
Northeast	19,919	13.4	3,931 (19.7)	
Place of residence				
Urban	51,133	34.4	3,520 (6.9)	<0.001
Rural	97,522	65.6	9,332 (9.6)	
Education				
No education	65,709	44.2	7,514 (11.4)	<0.001
Primary	23,584	15.9	1,466 (6.2)	
Secondary	48,785	32.8	2,665 (5.5)	
Higher	10,577	7.1	1,207 (11.4)	
Social group				
SC	31,139	22.0	2,846 (9.1)	<0.001
ST	12,919	9.1	1,530 (11.8)	
OBC	63,958	45.1	5,337 (8.3)	
Others	33,876	23.9	2,511 (7.4)	
Religion				
Hindu	123,425	83.1	9,536 (7.7)	<0.001
Muslim	16,831	11.3	2,580 (15.3)	
Christian	3,880	2.6	437 (11.3)	
Others	4,433	3.0	291 (6.6)	
Wealth				
Poorest	24,749	16.7	4,651 (18.8)	<0.001
Poorer	28,033	18.9	2,676 (9.5)	
Middle	30,845	20.8	1,915 (6.2)	
Richer	31,446	21.2	1,648 (5.2)	
Richest	33,581	22.6	1,962 (5.8)	
Mass Media				
Low	103,858	69.9	9,825 (9.5)	<0.001
Moderate	31,356	21.1	2,024 (6.5)	
High	13,440	9.0	1,003 (7.5)	
Relation to husband prior to marriage				
No	131,436	88.4	11,701 (8.9)	<0.001
Yes	17,183	11.6	1,147 (6.7)	
Age at first marriage ++				
<18	70,220	48.6	4,038 (5.8)	<0.001
18–21	38,825	26.9	2,764 (7.1)	
≥21	35,408	24.5	5,660 (16)	
Age at first sex ++				
<18 years	64,483	45.9	3,825 (5.9)	<0.001
18–21	44,200	31.5	3,474 (7.9)	
≥21	31,682	22.6	5,033 (15.9)	
Household structure ++				
Nuclear	84,608	56.9	8,089 (9.6)	<0.001
Non-nuclear	64,047	43.1	4,762 (7.4)	
Previous parity ++				
Zero	12,799	8.6	598 (4.7)	<0.001
1–2	89,563	60.3	4,119 (4.6)	
3–4	34,468	23.2	3,863 (11.2)	
5+	11,825	8.0	4,271 (36.1)	
Total	148,655	100	12,852 (8.7)	

Women aged 40–49 years were taken as an eligible sample for delayed motherhood for their last birth; total sample may or may not add to *N* because of missing values; 95% CI for national-level delayed motherhood ranges from 8.1% to 9.3%.

The results in Table 2 present the classical multilevel logistic regression analysis of individual/household and community-level factors associated with delayed motherhood in India. The result shows the significant state, district-level, and PSU-level variations in delayed motherhood, with model 4 being the best-fitted model considering its lowest value of AIC, Bayesian information criterion, and log-likelihood among all models. In model 1 (Null Model), the variance of the random factor for the state, district, and PSUs are 0.64, 0.19, and 0.36 respectively, showing heterogeneity in delayed motherhood at different levels. Because the variance and ICC (Supplementary Table A1) are greater than zero, it indicates that the multilevel analysis should be considered an appropriate approach for further analysis. In model 2, individual-level factors such as age, education, age at first marriage, age at first sex, and previous parity are significantly associated with delayed motherhood. Yet these factors are found to be significant after controlling the household-level factor in model 3 and the household/community-level factor in model 4. Model 4 shows that women with primary education (AOR: 0.91; CI: 0.85–0.98) were having a lower odds of delayed motherhood, whereas women with higher education (AOR: 2.0; CI: 1.80–2.23) were having a higher odds of delayed motherhood compared with the respondents with no formal education. The odds of delayed motherhood were 5.1 and 2.2 times higher among the women married or having sex after the age of 21 years in comparison with the women married or having sex before 18 years, respectively. More importantly, the likelihood of becoming a mother after the age of 35 rapidly increases with the number of prior parities. For instance, the likelihood of having delayed motherhood was 1.42 (CI: 1.29–1.56), 4.66 (CI: 4.20–5.17), and 24.74 (CI: 22.05–27.75) times higher for women with 1–2, 3–4, and 5+ prior parity, respectively. Muslims and Christians have a higher odds of delayed motherhood than Hindu women, respectively, by 62% (AOR: 1.62; CI: 1.50 −1.76) and 19% (AOR: 1.19; CI: 1.06 −1.34). In addition, the odds of becoming a mother after age 35 decrease rapidly with the increasing wealth of women. The null model (model 1) shows that 19% (ICC: 0.19; CI: 0.14–0.25) variation in the delayed motherhood is attributed to between-district variation, which further reduces to 6% (ICC: 0.06; CI: 0.04–0.09) in the full model (model 4) (Supplementary Table A1). This suggests that variations in clustering at the district level may be responsible for variations in the likelihood of delayed motherhood.

**Table 2:** Classical multilevel logistic regression analysis of individual/household and community-level factors associated with delayed motherhood in India.

Background variables	Model 1	Model 2	Model 3	Model 4
Individual-level factors	AOR [95% CI]	AOR [95% CI]	AOR [95% CI]	AOR [95% CI]
Age (ref: 40–44)				
45–49		1.37*** [1.32,1.43]	1.44*** [1.38,1.51]	1.44*** [1.38,1.50]
Education (ref: no education)				
Primary		0.80*** [0.75,0.85]	0.91** [0.85,0.97]	0.91** [0.85,0.98]
Secondary		0.76*** [0.71,0.80]	1.00 [0.94,1.07]	1.01 [0.94,1.07]
Higher		1.31*** [1.19,1.44]	2.0*** [1.79,2.22]	2.0*** [1.80,2.23]
Mass media (ref: low)				
Moderate		0.91** [0.85,0.96]	1.02 [0.96,1.09]	1.03 [0.96,1.09]
High		0.96 [0.88,1.05]	1.12* [1.02,1.23]	1.13* [1.02,1.24]
Relation to husband prior to marriage (ref: no)				
Yes		1.02 [0.95,1.10]	1.00 [0.93,1.08]	1.00 [0.93,1.08]
Age at first marriage (ref: <18years)				
18–21		1.57*** [1.44,1.70]	1.50*** [1.37,1.63]	1.50*** [1.37,1.63]
≥21		5.67*** [5.15,6.25]	5.14*** [4.65,5.67]	5.13*** [4.65,5.67]
Age at first sex (ref: <18 years)				
18–21		1.48*** [1.36,1.60]	1.48*** [1.36,1.61]	1.48*** [1.36,1.61]
≥21		2.14*** [1.94,2.36]	2.17*** [1.97,2.40]	2.18*** [1.97,2.41]
Previous parity (ref: zero)				
1–2		1.52*** [1.39,1.67]	1.42*** [1.29,1.57]	1.42*** [1.29,1.56]
3–4		5.52*** [4.99,6.10]	4.68*** [4.22,5.19]	4.66*** [4.20,5.17]
5+		31.70*** [28.36,35.42]	24.86*** [22.15,27.89]	24.74*** [22.05,27.75]
Household-level factors				
Place of residence (ref: urban)				
Rural			0.89*** [0.83,0.95]	0.88*** [0.82,0.94]
Social group (ref: ST)				
SC			0.95 [0.89,1.01]	0.94 [0.87,1.01]
OBC			0.87*** [0.82,0.93]	0.84*** [0.78,0.90]
Others			0.83*** [0.77,0.90]	0.80*** [0.73,0.87]
Religion (ref: Hindu)				
Muslim			1.63*** [1.51,1.76]	1.62*** [1.50,1.76]
Christian			1.19** [1.06,1.34]	1.19** [1.06,1.34]
Others			1.04 [0.92,1.17]	1.04 [0.92,1.18]
Wealth (ref: poorest)				
Poorer			0.61*** [0.57,0.65]	0.62*** [0.58,0.66]
Middle			0.47*** [0.44,0.51]	0.48*** [0.45,0.52]
Richer			0.38*** [0.35,0.41]	0.39*** [0.36,0.42]
Richest			0.33*** [0.29,0.36]	0.34*** [0.31,0.38]
Household structure (ref: nuclear)				
Non-nuclear			0.87*** [0.83,0.90]	0.86*** [0.83,0.90]
District-level factors				
Percentage of below primary education				1.00 [1.00,1.01]
Concentration of marginalized population (ST)				1.00 [1.00,1.00]
Percentage poverty				1.01** [1.00,1.01]
Random effect result				
Variance for state	0.64 [0.40, 1.04]	0.28 [0.17, 0.46]	0.15 [0.09, 0.26]	0.16 [0.09, 0.27]
VPC for state	0.14 [0.10, 0.21]	0.07 [0.05, 0.11]	0.04 [0.03, 0.07]	0.04 [0.03, 0.07]
Variance for districts	0.19 [0.16, 0.22]	0.11 [0.09, 0.13]	0.09 [0.07, 0.11]	0.08 [0.06, 0.1]
VPC for district	0.04 [0.04, 0.04]	0.03 [0.02, 0.03]	0.02 [0.02, 0.03]	0.02 [0.02, 0.03]
Variance for PSU	0.36 [0.32, 0.41]	0.18 [0.14, 0.24]	0.17 [0.12, 0.23]	0.17 [0.12, 0.23]
VPC for PSU	0.08 [0.08, 0.08]	0.05 [0.04, 0.06]	0.05 [0.03, 0.06]	0.05 [0.03, 0.06]
Model fitness				
Log-likelihood	−45,605.05	−34,843.16	−32,357.21	−32,346.42
AIC	91,218.11	69,722.32	64,772.42	64,756.84
BIC	91,257.74	69,899.18	65,056.00	65,069.75

VPC, variance partitioning coefficient; AIC, Akaike information criterion; BIC, Bayesian information criterion; PSU, primary sampling unit.

***P < 0.001.

*P < 0.01.

*P < 0.05.

**Figure 2:**
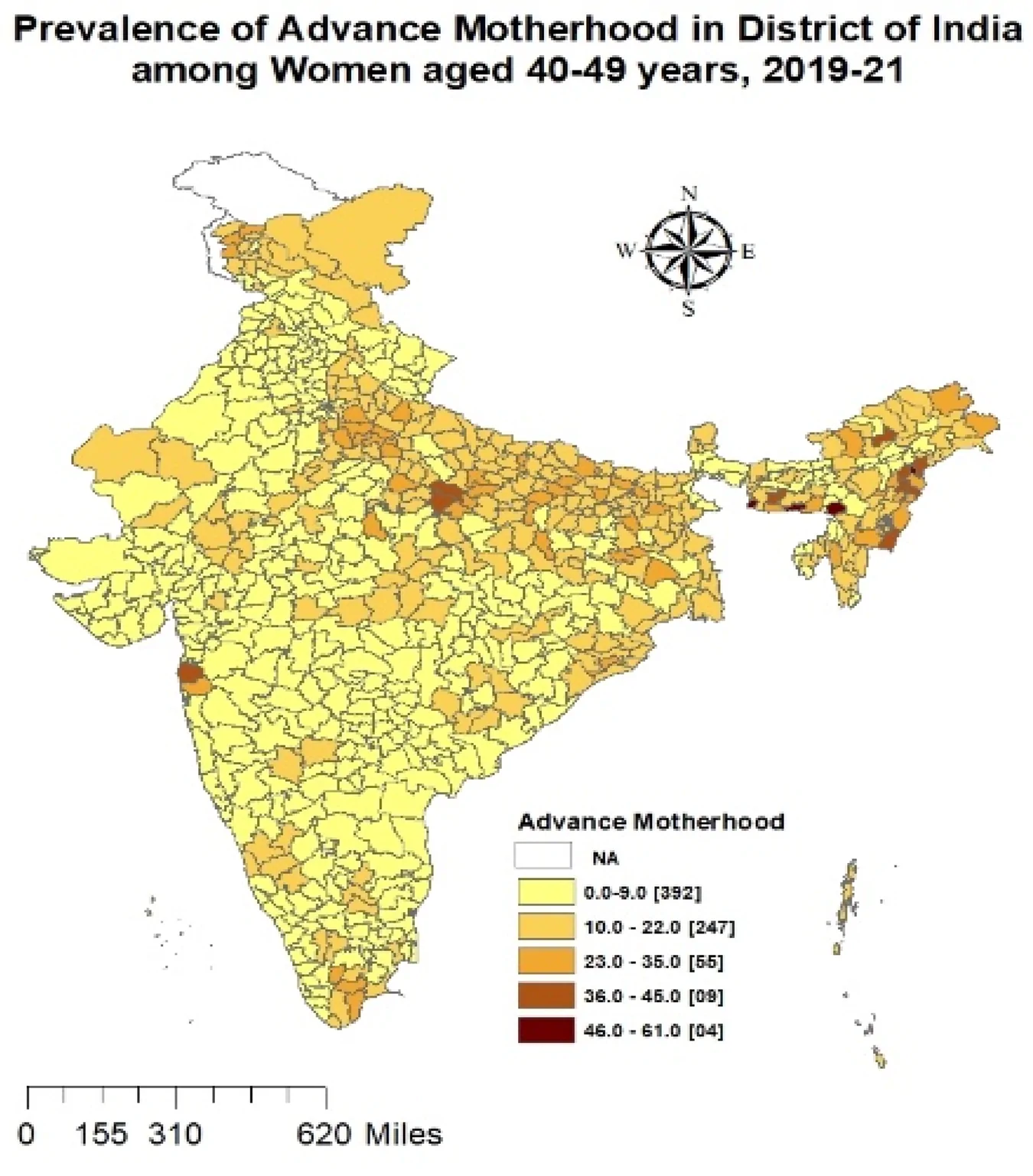
Percentage distribution of delayed motherhood in districts of India, 2019–2021.

Figure 2 demonstrates the district-level prevalence of delayed motherhood in India during 2019–21. The prevalence of delayed motherhood ranged from negligible levels in districts such as Jabalpur, Medchal-Malkajgiri, and Suryapet to 60.18% in the South West Khasi Hills district of Meghalaya. The national prevalence of delayed motherhood was 8.7% (95% CI: 8.1–9.3). More than half of the districts (55.4%) reported prevalence levels between 0% and 9%, including districts such as Palakkad (4.3%), Jaipur (5.2%), Surat (6.7%), and Pathankot (9.0%). In contrast, 55 districts reported prevalence levels between 23% and 35%, while 8 districts and 4 districts reported prevalence levels between 36% and 45% and 46% and 61%, respectively. Higher prevalence districts were predominantly concentrated in the northeastern region, particularly in Meghalaya, Assam, Manipur, and Nagaland, along with selected districts from eastern India. Conversely, lower prevalence districts were mainly observed across several northern, western, and southern states of the country.

**Figure 3:**
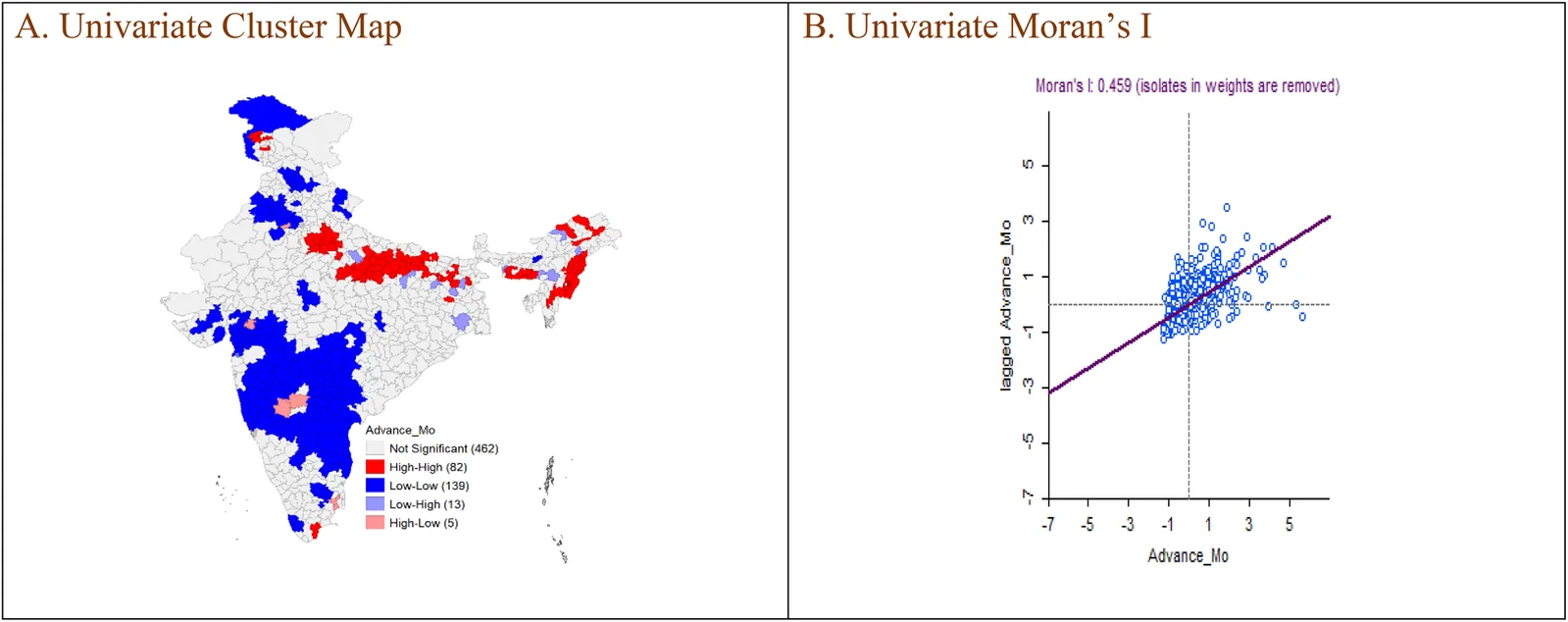
Univariate LISA cluster maps of India showing the geographic clustering (hotspots and cold spots) of delayed motherhood in Indian districts, 2019–2021. **(a)** A univariate cluster map and **(b)** univariate Moran’s *I*.

Table 3 and Figure 3 depicts univariate and bivariate Moran's *I* statistics showing the spatial dependence for the district-level prevalence of mesoscale variables with delayed motherhood in India, 2019–21. Univariate Moran's *I*-value for delayed motherhood (Moran's *I* = 0.459, *Z*-value: 17.77) and its cluster map (Figure 3B) indicate high spatial autocorrelation in delayed motherhood in the districts of India. The value of Empirical Bayes Bivariate Moran's *I* ranges from −0.001 for rural areas to 0.559 for women married after 21 years of age. Among the all mesoscale variables, first marriage after the age of 21 (Moran's *I*: 0.559, *Z*-value:21.38) followed by first sex above age 21 (Moran's *I*: 0.53, *Z*-value:20.92) and low mass media exposure (Moran's *I*: 0.468, *Z*-value:18.66), respectively, show the highest positive spatial autocorrelation with delayed motherhood. Figure 3A shows that the hotspot districts (high–high clusters) were predominantly observed in the northeastern states, including Assam, Arunachal Pradesh, Nagaland, Manipur, and Mizoram, as well as in parts of eastern and central India, particularly Bihar, Jharkhand, West Bengal, Odisha, and Chhattisgarh. In contrast, coldspot districts (low–low clusters) were mainly concentrated in the southern and western regions, including Karnataka, Maharashtra, Telangana, Andhra Pradesh, Gujarat, Rajasthan, and parts of Madhya Pradesh, Tamil Nadu, and Kerala.

**Table 3:** Univariate and bivariate Moran's *I* statistics showing the spatial dependence for the district-level prevalence of delayed motherhood in India, 2019–2021.

District-level percentage of mesoscale indicators/variables	Delayed motherhood (Univariate)		Delayed motherhood (bivariate)		Delayed motherhood (Empirical Bayes bivariate)	
	Moran's *I*-values	*Z*-value	Moran's *I*-values	*Z*-value	Moran's *I*-values	*Z*-value
Delayed motherhood	0.459	17.77	Delayed motherhood			
Rural	0.181	6.95	0.098	4.86	−0.001	0.03
No education	0.536	20.74	0.111	5.74	0.173	7.32
ST	0.414	16.56	0.158	7.71	0.435	15.55
Christians	0.409	16.15	0.184	8.67	0.311	6.98
Poorest	0.492	19.14	0.291	14.1	0.267	9.74
No prior relationship	0.637	24.75	0.215	10.78	0.441	17.12
Low mass media	0.457	18.03	0.077	3.98	0.468	18.66
First marriage above 21 years	0.545	20.86	0.091	4.7	0.559	21.38
First sex above 21 years	0.563	21.27	0.096	4.98	0.53	20.92
Previous parity more than 4	0.463	18.06	0.322	14.34	0.341	12.42

**Figure 4:**
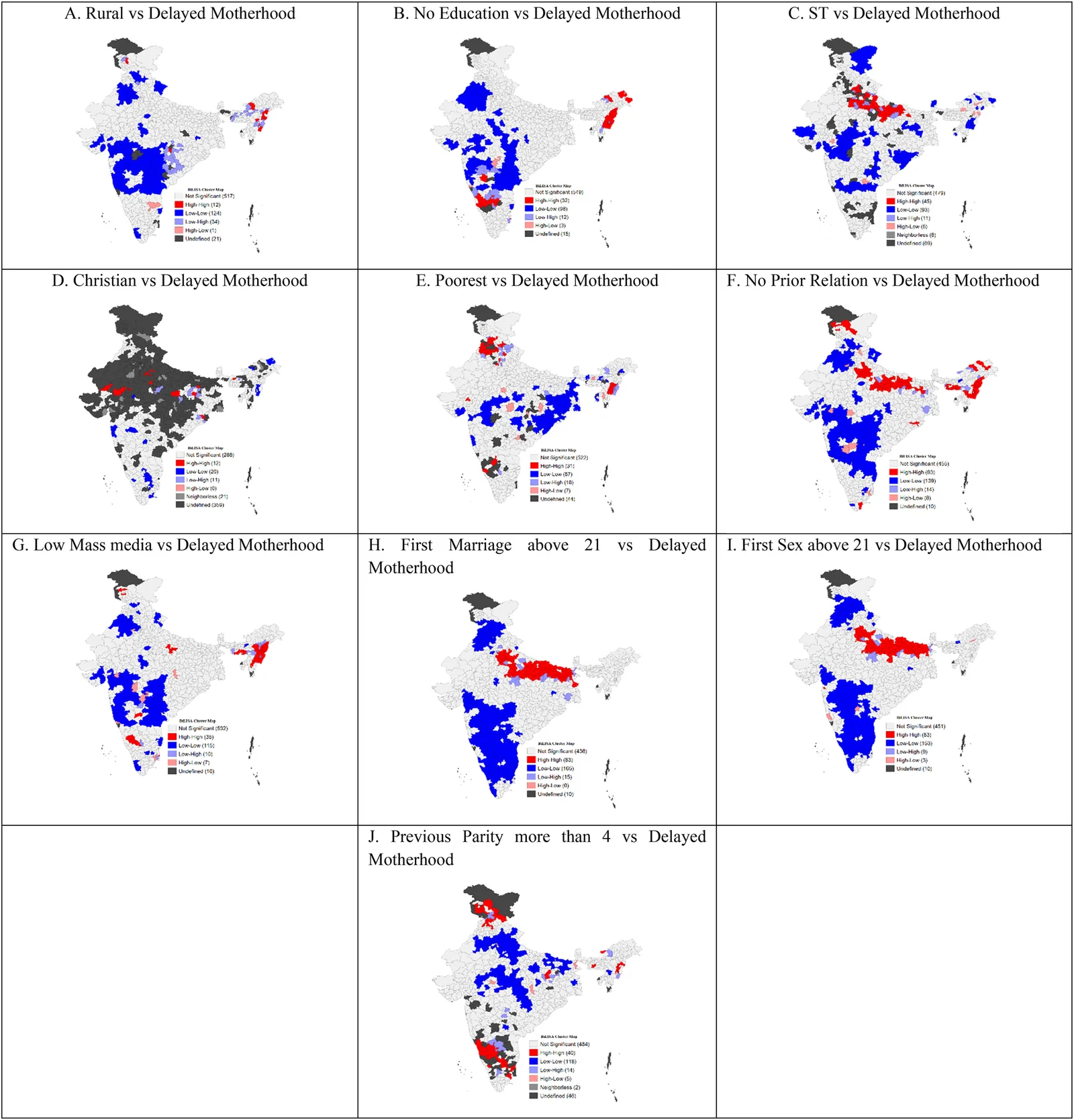
Empirical Bayes Bivariate LISA cluster maps of India showing the geographic clustering (hotspots and cold spots) of delayed motherhood in Indian districts, 2019–2021. **(A)** rural vs. delayed motherhood, **(B)** no education vs. delayed motherhood, **(C)** ST vs. delayed motherhood, **(D)** Christian vs. delayed motherhood, **(E)** poorest vs. delayed motherhood, **(F)** no prior relation vs. delayed motherhood, **(G)** low mass media vs. delayed motherhood, **(H)** first marriage above 21 vs. delayed motherhood, **(I)** first sex above 21 vs. delayed motherhood, and **(J)** previous parity more than 4 vs. delayed motherhood.

Figure 4 presents the Empirical Bayes Bivariate LISA cluster maps illustrating the spatial association between delayed motherhood in a given district and selected predictors in neighboring districts across India, 2019–2021. Map G indicates that 35 out of 707 districts (approximately 5%) exhibited high–high clusters, reflecting districts with a high prevalence of delayed motherhood surrounded by neighboring districts with a higher prevalence of low mass media exposure. Similarly, Map H shows that 83 districts (about 12%) formed high–high clusters, representing that areas with a high prevalence of delayed motherhood is spatially associated with neighboring districts having a high proportion of women marrying after the age of 21 years. Map I further shows that 153 districts were identified as low–low clusters for age at first sex ≥21 years, with many located in states such as Maharashtra, Karnataka, Andhra Pradesh, Tamil Nadu, Telangana, Kerala, Punjab, Haryana, and Himachal Pradesh. A similar spatial pattern was observed for previous parity of more than four children, with 40 districts (nearly 6%) forming high–high clusters and 118 districts (approximately 17%) forming low–low clusters. The corresponding bivariate significance maps are presented in Supplementary Figure F1, indicating the statistical significance of these spatial associations.

Table 4 presents the adjusted coefficients of the correlates associated with delayed motherhood in India during 2019–2021. Initially, an ordinary least-squares (OLS) regression model was employed without accounting for the spatial structure of the data to examine the association between delayed motherhood and district-level mesoscale correlates. The model provides estimates of coefficients, standard errors, and *p*-values. A positive coefficient indicates that an increase in the independent variable is associated with an increase in the dependent variable, whereas a negative coefficient suggests the opposite relationship. The results indicate that the coefficient for the rural population was 0.095; however, this association was not statistically significant. In contrast, women with no education showed a significant positive association with delayed motherhood (coefficient = 0.067). In addition, being Christian, belonging to the poorest wealth quintile, having age at first marriage above 21 years, age at first sexual intercourse above 21 years, and having a parity of more than four were all significantly and positively associated with delayed motherhood. These findings suggest that increases in these correlates are associated with higher levels of delayed motherhood.

**Table 4:** Result of regression analysis (OLS) showing the adjusted coefficients of the correlates for delayed motherhood in India, 2019–2021.

District-level mesoscale correlates	Delayed motherhood	
	Coef. (SE)	*P*-value
Rural	0.012 [0.009]	0.176
No education	0.067 [0.011]	0.000
ST	−0.006 [0.010]	0.554
Christians	0.072 [0.011]	0.000
Poorest	0.098 [0.011]	0.000
No prior relationship	−0.025 [0.015]	0.098
Low mass media	−0.020 [0.015]	0.167
First marriage above 21 years	0.244 [0.039]	0.000
First sex above 21 years	0.034 [0.038]	0.000
Previous parity more than 4	0.619 [0.021]	0.000
AIC value	3,893.87	
Log likelihood	−1,935.93	
Adjusted *R* square value	0.814	
No of districts	707	

The findings indicate that delayed motherhood is spatially clustered across districts in India, with significant positive spatial autocorrelation (Table 5). Among the spatial models, the SEM (Table 5) demonstrated better model fit compared with the SLM (Supplementary Table A2), as indicated by lower AIC and higher log-likelihood values. In the SEM, no education, Christian population, poorest wealth quintile, age at first marriage above 21 years, age at first sexual intercourse above 21 years, and higher parity (more than four children) were positively and significantly associated with delayed motherhood. Other variables, including rural residence, scheduled tribe population, no prior relationship, and low mass media exposure, were not statistically significant. The SLM results were largely consistent with those of the SEM. The spatial lag coefficient confirmed significant spatial dependence across districts. Similar to SEM, no education, Christian population, poorest wealth quintile, later age at first marriage, later age at first sexual intercourse, and higher parity remained significant positive correlates. In addition, rural residence showed a small positive association, while no prior relationship was negatively associated with delayed motherhood.

**Table 5:** Result of regression analysis (spatial error model) showing the adjusted coefficients of the correlates for delayed motherhood in India, 2019–2021.

District-level mesoscale correlates	Delayed motherhood	
	Coef. (SE)	*P*-Value
Rural	0.005 [0.008]	0.483
No education	0.042 [0.013]	0.001
ST	−0.001 [0.009]	0.936
Christians	0.054 [0.011]	0.000
Poorest	0.083 [0.012]	0.000
No prior relationship	−0.029 [0.018]	0.111
Low mass media	0.013 [0.015]	0.399
First marriage above 21 years	0.256 [0.037]	0.000
First sex above 21 years	0.023 [0.038]	0.000
Previous parity more than 4	0.615 [0.022]	0.000
Lambda value (lag coefficient)	0.433	
AIC value	3,814.760	
Log likelihood	−1,896.382	
Pseudo *R* square	0.843	
No of districts	707	

## 5 Discussion

The present study offers a spatially grounded and theoretically integrated examination of delayed motherhood in India, demonstrating that the postponement of first birth is neither spatially uniform nor socially neutral but instead deeply embedded within broader processes of demographic transition, modernization, gender transformation, and regional inequality. Consistent with classical demographic transition theory and the SDT framework, which link fertility postponement to rising education, urbanization, women's autonomy, and changing family norms (6, 7, 35, 36), the findings reveal that districts characterized by higher female educational attainment, greater urban concentration, stronger economic development, and improved access to health services exhibit a significantly higher prevalence of delayed motherhood. Conversely, districts where early marriage norms persist, female schooling remains limited, and socioeconomic deprivation is pronounced continue to show lower levels of postponement of first birth. This uneven transformation aligns with modernization perspectives that emphasize how structural changes in education systems, labor markets, and value orientations reshape reproductive behavior (8, 37). Recent empirical evidence from India further supports these structural shifts; for instance, a comprehensive joinpoint and age–period–cohort analysis documented a sustained upward shift in age at first birth across successive cohorts, highlighting generational changes in reproductive timing (12). Similarly, district-level multilevel and geospatial analyses of early marriage and motherhood across 707 districts revealed strong socioeconomic and contextual determinants shaping reproductive transitions (14), reinforcing the importance of localized structural conditions. Yet, the spatial analysis undertaken in the present study further demonstrates that these processes cluster geographically rather than diffuse uniformly across the country.

The presence of statistically significant spatial autocorrelation confirms that delayed motherhood practices are spatially patterned and influenced by geographic proximity, shared institutional contexts, and diffusion of social norms, consistent with foundational contributions in spatial econometrics (15, 16, 38). Such clustering suggests that districts are not independent demographic units but are embedded within broader regional systems where social change, policy implementation, and economic development spill across administrative boundaries. Although recent national-level evidence has documented temporal shifts in delayed motherhood (12), district-level spatial clustering observed in this study reveals how these trends manifest unevenly across space. Furthermore, earlier geospatial work on early marriage and motherhood (14) underscores how socioeconomic vulnerability and regional disadvantage remain concentrated in particular geographic pockets, which directly shape patterns of reproductive timing. Although national data from the National Family Health Survey (NFHS-5) document a gradual increase in age at first birth in India (1), district-level patterns uncovered in this study highlight substantial intrastate heterogeneity that remains obscured in aggregated statistics (10, 11). This geographic differentiation reinforces the need to move beyond generalized national narratives and instead interpret delayed motherhood within localized socioeconomic and cultural contexts.

State-level policy environments further help explain observed spatial variation. Investments in girls’ secondary and higher education, expansion of reproductive and maternal health services under the National Health Mission, and implementation of conditional cash transfer schemes aimed at promoting girls’ schooling and delaying marriage have contributed to shifts in reproductive timing (18, 19, 39). Evidence from district-level policy-sensitive analyses also suggests that contextual governance factors significantly mediate reproductive outcomes (14). The enforcement of the Prohibition of Child Marriage Act (2006) plays a critical role in shaping age at marriage and subsequent timing of first birth. States that demonstrate stronger governance capacity and more consistent policy implementation tend to show a higher prevalence of delayed motherhood, suggesting that institutional context mediates demographic change. At the same time, persistent sociocultural norms, kinship systems, and patriarchal gender structures in certain regions continue to constrain women's reproductive autonomy, reflecting the coexistence of modern and traditional family systems within India (40). These findings underscore that demographic transition in the country is not linear but layered, with advanced and traditional fertility regimes operating simultaneously across space—a pattern also reflected in cohort-based temporal analyses (12).

The strong association between female education and delayed motherhood observed in this study aligns with extensive empirical evidence demonstrating that education enhances women's decision-making autonomy, labor force participation, aspirations for upward mobility, and bargaining power within households (13, 41). Urbanization, media exposure, and economic diversification further reinforce postponement by reshaping normative expectations regarding marriage and family formation. Recent socioeconomic shifts including expansion of higher education, rising costs of childrearing, increased female employment aspirations, and changing intergenerational expectations have contributed to delayed childbearing particularly in metropolitan and economically dynamic districts (9, 42), consistent with observed period and cohort effects in recent national analyses (12). However, the persistence of low delayed-motherhood prevalence in certain clusters underscores enduring spatial inequalities in educational access, labor market opportunities, and health infrastructure, echoing earlier geospatial findings on concentrated early motherhood (14).

By integrating spatial econometric techniques with demographic analysis, this study advances existing literature in several important ways. Although previous research has documented temporal trends using age–period–cohort modeling (12) and mapped determinants of early motherhood through multilevel geospatial frameworks (14), the present analysis explicitly models spatial dependence and geographic clustering in delayed motherhood, thereby providing deeper insights into how modernization, gender empowerment, and institutional variation intersect across districts. The policy implications of these findings are substantial. Districts identified as low-prevalence clusters require intensified investments in girls’ secondary and higher education, stricter enforcement of child marriage legislation, improved reproductive health awareness, and expansion of adolescent health services. Meanwhile, districts experiencing rising delayed motherhood may require strengthened maternal healthcare systems responsive to older first-time mothers, as medical literature documents potential obstetric risks associated with postponed childbearing (35). Recognizing spatial clustering also suggests that regionally coordinated interventions may generate spillover benefits across neighboring districts. Overall, delayed motherhood in India emerges as a spatially uneven manifestation of modernization, socioeconomic mobility, gender transformation, and differential policy implementation. Sustaining equitable progress will therefore require spatially sensitive, institutionally grounded, and region-specific strategies that address both structural inequalities and evolving sociocultural norms shaping reproductive decision-making (35, 43).

## 6 Conclusions

This study shows that delayed motherhood in India is a spatially differentiated demographic process shaped by district-level socioeconomic conditions, cultural norms, and state policy environments rather than a uniform national trend. The presence of spatial clustering indicates that reproductive behaviors are influenced by regional development patterns and spillover effects across neighboring districts. Therefore, policy responses must move beyond generalized national strategies and adopt context-specific, multiscalar approaches. At the national and state levels, strengthening girls’ education, enforcing child marriage legislation, and expanding reproductive health services remain critical. At the district level, targeted interventions should focus on identified low-prevalence clusters, while districts experiencing rising delayed motherhood require responsive maternal healthcare systems for older first-time mothers. Incorporating spatial analytics into policy planning will enable more efficient resource allocation and promote equitable demographic transition across regions.

### 6.1 Strengths

This study has several notable strengths. First, it utilizes nationally representative data from the National Family Health Survey (NFHS-5), which provides large-scale, high-quality, and district-level information across India, ensuring strong external validity and generalizability of the findings. Second, by employing spatial analytical techniques alongside conventional regression methods, the study moves beyond traditional demographic analysis to explicitly examine geographic clustering, spatial autocorrelation, and regional disparities in delayed motherhood. This spatially informed approach allows identification of high- and low-prevalence clusters, thereby uncovering intrastate heterogeneity that is often masked in aggregated national or state-level analyses. Third, the integration of theoretical perspectives, demographic transition theory, modernization theory, and gender empowerment frameworks provides a coherent conceptual basis for interpreting the findings within broader socioeconomic and cultural transformations. Fourth, the district-level focus strengthens policy relevance by generating context-specific insights that can inform decentralized planning and regionally targeted interventions.

### 6.2 Limitations

The cross-sectional design of NFHS-5 limits the ability to establish causal relationships between socioeconomic factors and delayed motherhood; associations observed in the analysis should therefore be interpreted cautiously. Second, the reliance on self-reported age at first birth may be subject to recall bias, particularly among older respondents. Third, although spatial econometric techniques account for geographic dependence, unobserved contextual variables, such as local governance quality, cultural norms, or implementation intensity of state-level policies, could not be fully captured because of data constraints. Fourth, the analysis is restricted to available survey indicators and may not fully incorporate life-course dynamics, employment trajectories, or qualitative dimensions of reproductive decision-making. Finally, while district-level analysis improves granularity, it may still conceal microlevel heterogeneity within districts, particularly in socially stratified settings.

### 6.3 Paper context

*Main findings*: The trend of delayed motherhood, with women becoming mothers after age 34, is rising because of societal changes and evolving gender roles. Higher education and marriage after age 21 significantly increase the odds of delayed motherhood. The prevalence rate varies from 0% to 60.18% across districts, with education being a strong predictor.

*Added knowledge*: This study provides new insights into the distribution and determinants of delayed motherhood in India, and the research identifies significant predictors of delayed motherhood at multiple levels—state, district, and PSU. The findings highlight the strong influence of higher education and later marital age on the likelihood of delayed motherhood.

*Global health impact for policy and action*: This research highlights the critical impact of education and marital age on delayed motherhood. Addressing these issues through targeted policies and services is crucial for improving maternal and child health. Effective governance can ensure that the needs of women experiencing delayed motherhood are met, benefiting the entire population.

## Data Availability

Publicly available datasets were analyzed in this study. These datasets can be found here: NFHS data are a nationally representative dataset that is freely available in the public domain. For more details, visit www.measuredhs.com and can be accessed after the request is made to and approved by the DHS program.
