# Supplementary material for: Geographical clustering of delayed motherhood in India: an application of multilevel and geospatial analyses
Source: Front Glob Womens Health. 2026 Aug 17;7:1762981. doi: 10.3389/fgwh.2026.1762981 (PMC13526969; doi:10.3389/fgwh.2026.1762981)
Supplement: Supplementary file 1 [file Supplementaryfile1.docx]

**Supporting Information’s**

**Appendix Tables:**

**Table A1:** Clustering of delayed Motherhood at state, district and PSU level in India 2019-21.

| **Level** | **ICC (95% CI) Model 1** | **ICC (95% CI) Model 2** | **ICC (95% CI) Model 3** | **ICC (95% CI) Model 4** |
| --- | --- | --- | --- | --- |
| **State** | 0.14 [0.09 0.21] | 0.07 [0.04 0.11] | 0.04 [0.02 0.07] | 0.04 [0.02 0.07] |
| **District** | 0.19 [0.14 0.25] | 0.10 [0.07 0.14] | 0.06 [0.05 0.09] | 0.06 [0.04 0.09] |
| **PSU** | 0.27 [0.22 0.32] | 0.15 [0.12 0.18] | 0.11 [0.09 0.14] | 0.11 [0.09 0.14] |

Notes: ICC: intra-cluster correlation coefficient, CI: confidence interval, PSU: primary sampling unit

**Figure F1:** Emp. Bayes Bivariate LISA significance maps of India showing the geographic significance of delayed motherhood in Indian districts, 2019-21.

| A. Rural vs Delayed Motherhood   | B. No Education vs Delayed Motherhood   | C. ST vs Delayed Motherhood   |
| --- | --- | --- |
| D. Christian vs Delayed Motherhood   | E. Poorest vs Delayed Motherhood   | F. No Prior Relation vs Delayed Motherhood   |
| G. Low Mass media vs Delayed Motherhood   | H. First Marriage above 21 vs Delayed Motherhood   | I. First Sex above 21 vs Delayed Motherhood   |
|  | J. Previous Parity more than 4 vs Delayed Motherhood   |  |

**Table A2:** Result of regression analysis (Spatial Lag Model) showing the adjusted coefficients of the correlates for delayed Motherhood in India, 2019-21

| District level meso scale correlates | Delayed Motherhood | |
| --- | --- | --- |
|  | Coef. (SE) | P Value |
| Rural | 0.016 [0.008] | 0.047 |
| No Education | 0.052 [0.  011] | 0.000 |
| ST | -0.004 [0.009] | 0.686 |
| Christians | 0.061 [0.01] | 0.000 |
| Poorest | 0.079 [0.011] | 0.000 |
| No Prior Relationship | -0.046 [0.015] | 0.002 |
| Low Mass Media | -0.011 [0.014] | 0.441 |
| First Marriage above 21 Years | 0.25 [0.014] | 0.000 |
| First Sex above 21 Years | 0.008 [0.036] | 0.000 |
| Previous Parity more than 4 | 0.580 [0.020] | 0.000 |
| Rho Value (Lag coefficient) | **0.207** | |
| AIC value | **3828.970** | |
| Log Likelihood | **-1902.49** | |
| Pseudo R Square | **0.835** | |
| No of District | **707** | |
